# Undergraduate pharmacy student research experience—a win-win situation

**DOI:** 10.1177/17151635251380856

**Published:** 2025-11-05

**Authors:** Ryan R. D. Chan, Katelyn Halpape

**Affiliations:** College of Pharmacy and Nutrition, University of Saskatchewan, Saskatoon, SK; College of Pharmacy and Nutrition, University of Saskatchewan, Saskatoon, SK

## Introduction

As pharmacy researchers, we find ourselves at a unique crossroads of curiosity, imposter syndrome, and growth. We strive to address existing gaps in the literature and make meaningful contributions to patient care and pharmacy practice, all while developing our research skills. This article highlights the key “aha” moments from our research journeys and explores the reciprocal student–supervisor relationship that has supported our research successes. We offer perspectives from both an undergraduate pharmacy researcher (R.R.D.C.) and a junior faculty member in pharmacy (K.H.).

## Strengthening content knowledge

Ryan R. D. Chan (R.R.D.C.): Conducting research throughout my undergraduate training enhanced my understanding of pharmacotherapeutics by identifying and supplementing knowledge gaps with further exploring the “how” and “why.” Unlike the passive process of rote memorization required in didactic coursework, research empowered me to explore disease states and accompanying treatment guidelines through an evidence-based and critical thinking lens. For example, while completing a project on potential risk factors of venous thromboembolism (VTE) in psychiatric inpatients, I found myself applying Virchow’s Triad to rationalize how psychiatric inpatients may be at risk of VTE, which took my understanding of the topic to a much deeper level.^1^

Delving into the “how” and “why” of complex topics not only strengthened my understanding of pharmacotherapy but also deepened my appreciation for the research process, from idea generation to final publication, which has been pivotal to my growth as an aspiring clinician-researcher. Through undergraduate research training, I learned to formulate research questions that address meaningful gaps in pharmacy practice as well as design and conduct studies to explore these questions, including critically appraising existing literature. For example, in my third year, I proposed a project idea to 2 faculty members and subsequently completed a directed studies research elective involving a scoping review on take-home naloxone access and use among older adults living with pain.^2^ These experiences not only enhanced my research skills and content knowledge but also laid a strong foundation for future scholarly pursuits, including graduate studies and, one day, a position in academia.

Katelyn Halpape (K.H.): It has been rewarding to be part of R.R.D.C.’s research journey and to witness those lightbulb moments when complex concepts clicked. Supporting students as they connect theory to real-world application is one of the most fulfilling aspects of mentorship. For me, engaging in research and teaching at both the undergraduate and graduate levels continually deepens my understanding of pharmacotherapy and related content. These experiences reinforce that learning is a lifelong process.

## Growth mindset

R.R.D.C.: Experiencing rejection in academia, including manuscript submissions, grant proposals, or even ghosted email follow-ups, has impacted my perspective on failure and growth. While rejections are discouraging, I gradually adopted the philosophy instilled by my supervisor (K.H.) that “rejection equals redirection,” which transformed my approach from viewing setbacks as personal failures to instead seeing them as opportunities. For instance, an unsuccessful manuscript submission contributed to enhancing the clarity of our future submitted work. Similarly, a rejected grant proposal contributed to the successful receipt of future funding after implementing feedback and revising the project proposal. Ultimately, changing my perspective has nurtured curiosity, creativity, and resilience, turning the fear of failure into a more optimistic and proactive approach to facing future challenges.

K.H.: A growth mindset is essential for success in academia. By success, I mean not only achieving career milestones, such as tenure and promotion, but also maintaining personal well-being in the face of challenges. As R.R.D.C. has highlighted, a growth mindset is critical in academic environments, where feedback on grants, manuscripts, and case files is frequent. The ability to approach feedback with an open mind, critically evaluate it, and integrate constructive elements is crucial for both professional development and sustaining a healthy, resilient outlook.

## Imposter syndrome

R.R.D.C.: Working closely with accomplished academics can elicit unintentional feelings of imposter syndrome: a persistent fear of inadequacy and anxiety about disappointing one’s supervisor. I experienced this firsthand when I started as a research assistant with K.H. Despite her unwavering support and approachability, I felt intimidated and second-guessed every decision I made, worried that my inexperience or lack of clinical knowledge might reflect poorly on me. Over time, I came to understand that these feelings are a natural part of stepping outside one’s comfort zone and trying something new. Her encouragement fostered a supportive environment that enabled me to become more confident in my role. Acknowledging that imposter syndrome is a natural response to new challenges helped me shift my mindset, approach research with greater resilience, and fully embrace the learning process.

K.H.: Imposter syndrome is a reality in academia, even for those who may have been in the field for years. It is essential to acknowledge that feeling uncertain or uncomfortable is a natural part of embracing new challenges. Trusting in one’s skills and accepting that discomfort is a natural part of learning can make the process more manageable. Reaching out for support and setting realistic goals are also important, and I frequently remind learners that “it is okay not to have all the answers right away.”

## Mentorship

R.R.D.C.: Mentorship has contributed to our student–supervisor relationship through building trust, demonstrating resilience, and empowering one another. K.H. described mentorship to me as akin to a marathon, not a sprint; it requires patience, consistency, and commitment to the reciprocal growth and learning. Her approach to facilitating our mentorship includes leading by example and fostering a supportive environment that embodies gradual progression and continuous encouragement.

K.H.: I agree with R.R.D.C.’s reflection. Mentorship is indeed a marathon, requiring time, vulnerability, and a shared commitment to growth. Clear communication and setting a schedule for regular check-ins are essential to a successful mentorship, like following a training plan for a marathon. Just as athletes outline their goals and consult with coaches, mentees benefit from a structured approach, ongoing dialogue, and guidance along the way. I aim to provide this by developing structured work plans for projects involving learners. These include a schedule of project deadlines, regular check-in meetings, and an activity record where students can document their progress, reflect on their work, and jot down any questions they have. This allows us to easily track their development and learning over time.

## Career building

R.R.D.C.: Pursuing research as an undergraduate student has enhanced my critical thinking, communication, and collaboration skills. For instance, I have had the opportunity to present our research findings at local, provincial, national, and international conferences. K.H. has consistently empowered me to approach each presentation as an “opportunity to practice,” which has improved my confidence, tailored my presentations to specific audience members, and refined my 3-minute pitch.

K.H.: Mentorship is a 2-way learning experience that not only supports the mentee’s growth but also provides the mentor an opportunity to reflect on their strengths and areas for improvement. Guiding students like R.R.D.C. has helped me better understand my approach to communication, leadership, and teaching. Each mentorship experience encourages me to grow alongside my students and continuously evolve as an academic and educator.

## Paying it forward

R.R.D.C.: As I continue growing in my own research capabilities, I have had the privilege of supporting other learners embarking on their own research journeys, which has been a rewarding experience of giving back to the academic community. Ultimately, my goal is to continue paying it forward by sharing the pearls of wisdom instilled by mentors and, hopefully, inspiring the next generation of aspiring clinician-researchers.

K.H.: Much of what I have learned as a professional has come through experience and the support of exceptional mentors. Throughout my training and in my current role as a faculty member, I have been fortunate to be guided by health care professionals and educators who have helped shape the clinician, educator, and researcher I am today. Their time, energy, and encouragement provided a strong foundation that I now strive to build upon by supporting and mentoring pharmacy learners myself.

For me, paying it forward means more than sharing knowledge; it’s about creating a space where learners feel supported, challenged, and empowered. One of the most important steps I take at the beginning of a mentoring relationship is to get to know each student’s learning style, how they respond to feedback, and what they hope to gain from the experience. Setting clear expectations early on and revisiting them as needed helps to ensure that we are aligned and working toward shared goals.

I have also learned the value of flexibility. Not every learner is the same, and adapting my approach to suit their needs while maintaining open communication has allowed me to make the most of each mentoring relationship. My style continues to evolve, informed by the lessons I receive from my mentors as well as my experiences mentoring others. In this way, I hope to honour their influence and continue the cycle of growth and support within the pharmacy profession. I also want to instill in my mentees a spirit of paying it forward, so they feel inspired to do the same throughout their careers.

## Conclusion

Our shared experiences as a student and faculty member underscore the mutual growth of engaging in undergraduate pharmacy research. Overall, we have built a dynamic, reciprocal relationship rooted in trust, open-mindedness, and shared learning. Research has not only advanced our academic and professional development but has also deepened our commitment to enhancing patient care and contributing to the advancement of the pharmacy profession. We hope our reflections encourage others to invest in these enriching experiences and to recognize that when mentorship and research align, everyone wins. ■
